# Supplementary material for: Biological and Chemical Characterization of Musa paradisiaca Leachate
Source: Biology (Basel). 2023 Oct 11;12(10):1326. doi: 10.3390/biology12101326 (PMC10604775; doi:10.3390/biology12101326)
Supplement: Supplementary file 1 [file biology-12-01326-s001.zip › biology-2619228-supplementary.pdf]

Supplementary materials

Table S1. Monoclonal antibodies used in immunocytochemical characterization of glycopolymers in tomato seedlings and plants under control and drought stress.

**Glycopolymer**		**mAb**	**Epitope**	**References**
Pectins	Homogalacturonan	LM19	Homogalacturonan with low degree of esterification	[68]
		LM20	Homogalacturonan with high degree of esterification	
	Rhamnogalacturonan I	LM5	[β(1-4)galactan]>3 Rhamnogalacturonan I	[69]
		LM6	[α(1-5) highly branched arabinan]5/6 Rhamnogalacturonan I	[70]
		LM16	B-1,3-linked galactan backbone when substituted with a single β-1,6-linked Gal	[68]
	Xylogalacturonan	LM8	Xylogalacturonan associated with cell detachment and separation	[71]
Hemicelluloses	Xyloglucan	LM15	Xylosylated xyloglucan (XXXG)	[72]
		LM25	Xylosylated/galactosylated xyloglucan (XXLG and XLLG)	[73]
	Xylan	LM10	Unsubstituted and relatively low-substituted xylan	[74]
	Heteromanan	LM21	β(1-4) linked mannan from DP2 to DP5	[75]
	Arabinogalactan protein	JIM13	βGlcA-(1,3)-αGalA-(1,2)-Rha	[76,77]
	Extensin	LM1	unknown	[78]

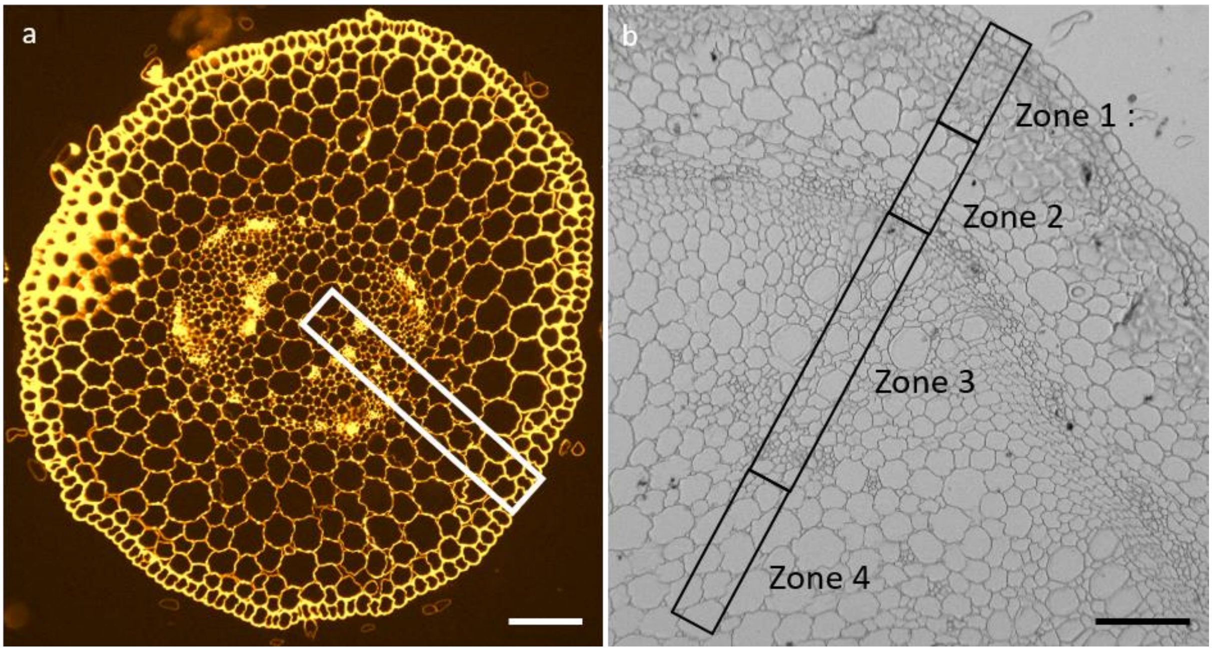

Figure S1. Region of interest (ROI) defined for pictures from *in vitro* and phytotron assays. a) *In vitro* assay : cross section of 13-day-old stem tomato seedling. The defined ROI contain all the tissues. b) Phytotron assay : cross section of 45-day-old stem tomato plant. Four ROI were defined as followed : zone 1 : epidermis + peridermis + collenchyma ; zone 2 : cortical parenchyma ;zone 3 : sclerenchyma + xylem + phloem ; zone 4 : medullar parenchyma. Scale bar : 100µm

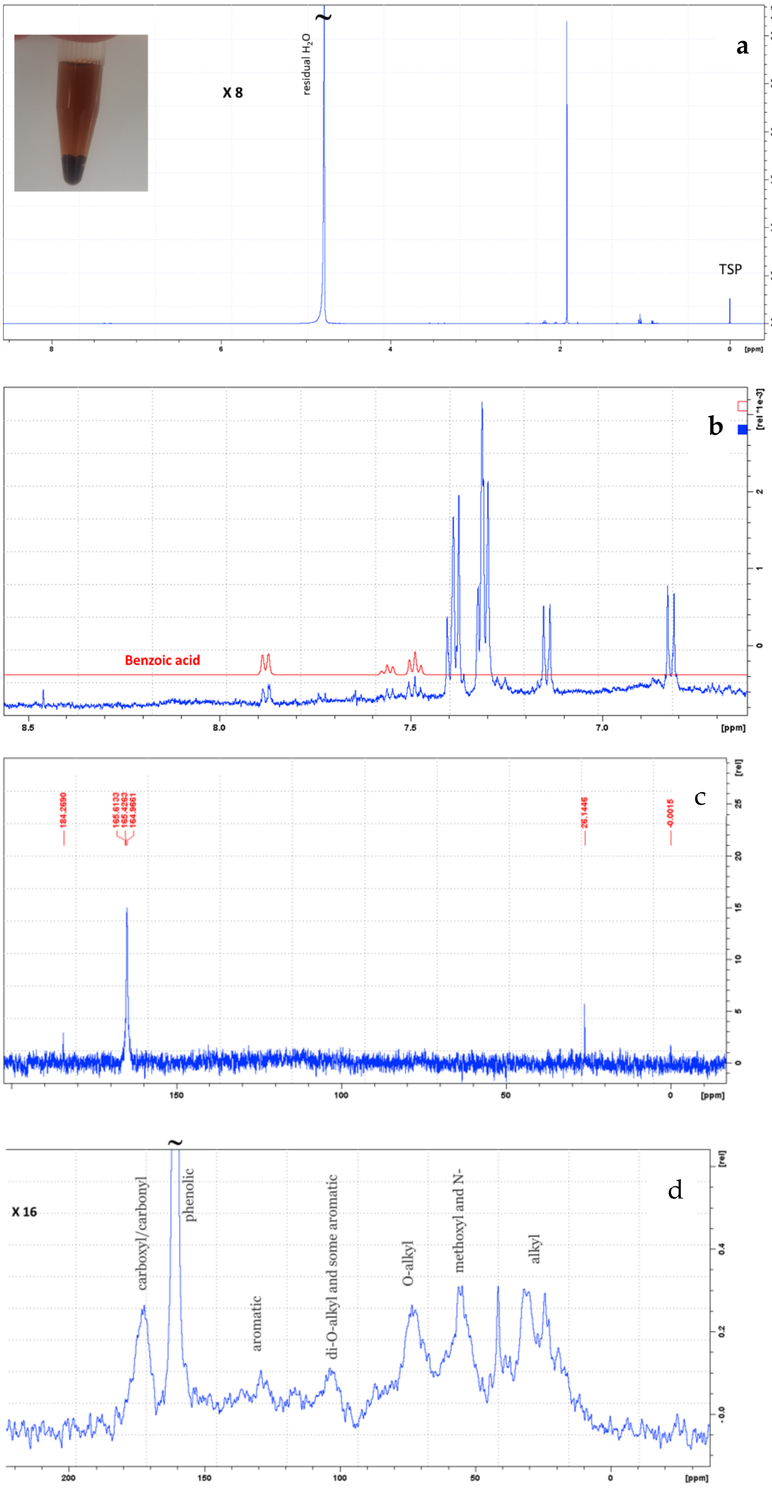

Figure S2. Characterization of plantain rachis leachate by liquid-state and solid-state NMR. a) native plantain rachis leachate by ^1H liquid-state NMR spectrum at 500 MHz. Zoom X8; b) Zoom in aromatic region. Comparison with ^1H NMR spectrum of benzoic acid (commercial compound) in D_2O pH 6.0 at 500 MHz; c) ^{13}C NMR spectrum of native plantain rachis leachate at 500 MHz; d) Solid-state $^1H/^{13}C$ MAS NMR spectrum of freeze-dried leachate at 400 MHz. Zoom X16. Putative annotation according to Preston et al.[35].

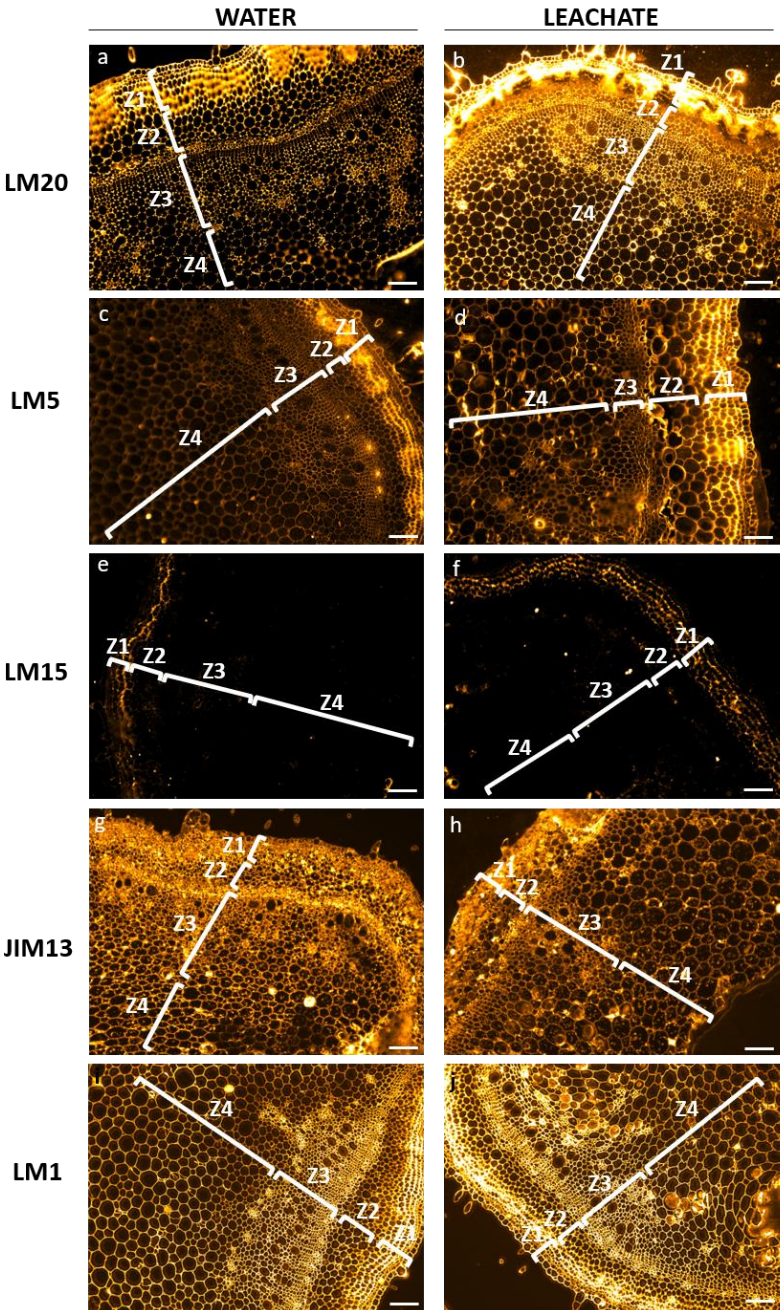

Figure S3. Significant immunocytochemical characterization of aerial parts of tomato plant after treatments with leachate and water (according to Mann-Whitney test). LM20, LM5, LM15, JIM13 and LM1: primary antibodies against glycopolymers epitopes.

			In vitro assay		
			Control	Leachate	pvalue
Pectins	Homogalacturonan	LM19	461.5 ±119.1	397.6 ±65.7	0.279
		LM20	1101.9 ±505.8	1332.0 ±579.1	0.516
	Rhamnogalacturonan I	LM5	644.8 ±67.9	702.0 ±135.8	0.569
		LM6	447.4 ±49.1	481.8 ±30.2	0.279
	Xylogalacturonan	LM8	371.9 ±46.2	406.8 ±127.3	0.871
Hemicelluloses	Xyloglucan	LM15	429.7 ±55.5	492.7 ±193.6	0.589
		LM25	502.6 ±61.9	493.1 ±50.7	0.626
	Xylan	LM10	412.6 ±45.7	406.5 ±39.4	0.663
	Heteromanan	LM21	384.4 ±31.4	424.3 ±122.3	0.957
Hydroxyproline rich glycoproteins (HRGP)	Arabinogalactan protein	JIM13	720.8 ±89.7	803.2 ±237.2	0.914
		LM16	346.7 ±12.4	356.6 ±29.4	0.845
	Extensin	LM1	380.1 ±30.3	425.7 ±64.2	0.279

			Phytotron assay											
			Z1			Z2			Z3			Z4		
			Control	Leachate	pvalue	Control	Leachate	pvalue	Control	Leachate	pvalue	Control	Leachate	pvalue
Pectins	Homogalacturonan	LM19	294.2 ±71.2	329.7 ±190.3	0.787	213.2 ±27.6	202.7 ±61.0	0.516	271.1 ±88.8	284.4 ±121.3	0.746	163.7 ±27.4	171.2 ±47.4	0.516
		LM20	897.9 ±367.2	985.6 ±634.6	0.805	409.9 ±100.9	489.9 ±250.9	0.671	292.3 ±80.6	414.4 ±54.3	0.020	279.9 ±91.6	362.8 ±142.9	0.376
	Rhamnogalacturonan I	LM5	942.9 ±108.3	1075.1 ±105.6	0.045	418.8 ±58.5	480.1 ±51.4	0.160	474.1 ±51.8	501.6 ±63.3	0.516	294.8 ±46.8	316.3 ±38.7	0.387
		LM6	1922.9 ±354.9	1952.4 ±367.7	1.000	858.1 ±137.0	965.0 ±311.6	0.596	1231.2 ±220.9	1510.5 ±643.4	0.724	646.2 ±75.8	694.3 ±141.5	0.548
	Xylogalacturonan	LM8	252.4 ±79.3	233.8 ±50.9	0.808	166.9 ±27.1	190.1 ±34.6	0.230	169.8 ±26.8	197.2 ±49.6	0.330	141.4 ±11.5	186.6 ±70.7	0.214
Hemicelluloses	Xyloglucan	LM15	294.8 ±86.3	457.8 ±108.5	0.045	185.9 ±38.0	243.4 ±68.3	0.045	225.4 ±68.8	270.1 ±103.9	0.482	146.7 ±6.8	178.1 ±56.4	1.00
		LM25	441.8 ±70.5	534.1 ±168.1	0.589	242.7 ±20.6	283.5 ±43.5	0.176	324.7 ±29.5	348.0 ±69.9	0.914	158.1 ±13.8	185.9 ±35.0	0.144
	Xylan	LM10	ND	ND	ND	ND	ND	ND	ND	ND	ND	ND	ND	ND
	Heteromanan	LM21	217.7 ±17.1	230.1 ±42.3	0.922	159.7 ±15.7	173.1 ±33.9	0.433	187.0 ±21.7	165.1 ±8.1	0.327	137.7 ±5.3	142.6 ±10.8	0.433
Hydroxyproline rich glycoproteins (HRGP)	Arabinogalactan protein	JIM13	419.9 ±80.9	658.3 ±78.6	0.011	385.5 ±46.4	313.8 ±28.5	0.013	421.1 ±84.2	431.5 ±70.5	0.914	234.5 ±79.0	239.7 ±41.1	0.387
		LM16	ND	ND	ND	ND	ND	ND	ND	ND	ND	ND	ND	ND
	Extensin	LM1	580.1 ±115.2	656.0 ±153.0	0.266	451.4 ±76.9	447.4 ±65.9	0.923	473.7 ±40.8	564.3 ±29.8	0.040	367.8 ±50.9	373.2 ±40.5	0.480

Figure S4. Effect of leachate application under drought stress on cell wall imaging in *in vitro* and phytotron assays. Values (in arbitrary unit) in squares correspond to leachate or control induced increase or decrease in glycopolymers epitopes detection. The p-value is referred according to the Mann-Whitney Test. The color squares differ significantly based on the Mann-Whitney Test. In green a significant increase compared to the control; in red a significant decrease compared to the control; in gray not significant. (Z1) zone 1: epidermis + peridermis + collenchyma; (Z2) zone 2: cortical parenchyma; (Z3) zone 3: sclerenchyma + xylem + phloem; (Z4) zone 4: medullar parenchyma. Each condition was replicated was replicated in 3 independent biological replicates. ND: not detected.

Supplemental data

ImageJ macro developed for fluorescence measurements :

```
open();
path = getDirectory("image");
nomLong=getTitle();
nomCourt=File.getNameWithoutExtension(path+nomLong);
getDimensions(width, height, channels, slices, frames);
getPixelSize(unit, pixelWidth, pixelHeight);
if (slices>1) {
   run("Z Project...", "projection=[Average Intensity]");
}

run("Enhance Contrast", "saturated=0.35");
//run("Z Project...", "projection=[Sum Slices]");
makeLine(460, 712, 3844, 2008);
waitForUser("Settings", "Please adjust the line to the region you need\nand be sure it is wide enough.\n\nThen, press OK");

// Get profile and display values in "Results" window
run("Clear Results");
profile = getProfile();
for (i=0; i<profile.length; i++){
   setResult("Distance", i, i*pixelWidth);
   setResult("Value", i, profile[i]);
   updateResults;
}

saveAs("Results", path+nomCourt+".csv");


   // Plot profile

Plot.create("Profile", "X", "Value", profile);

run("Close All");
selectWindow("Results");
run("Close");
```
